# Electrocardiographic predictors of successful resynchronization of left bundle branch block by His bundle pacing

**DOI:** 10.1111/jce.14845

**Published:** 2021-01-04

**Authors:** Ahran D. Arnold, Matthew J. Shun‐Shin, Daniel Keene, James P. Howard, Ji‐Jian Chow, Elaine Lim, Smaragda Lampridou, Alejandra A. Miyazawa, Amal Muthumala, Mark Tanner, Norman A. Qureshi, David C. Lefroy, Michael Koa‐Wing, Nick W. F. Linton, Phang Boon Lim, Nicholas S. Peters, Prapa Kanagaratnam, Angelo Auricchio, Darrel P. Francis, Zachary I. Whinnett

**Affiliations:** ^1^ National Heart and Lung Institute Imperial College London, Hammersmith Hospital London UK; ^2^ Cardiology Department North Middlesex University Hospital NHS Trust London UK; ^3^ Cardiology Department St. Bartholomew's Hospital, Barts Health NHS Trust London UK; ^4^ Division of Cardiology Fondazione Cardiocentro Ticino Lugano Switzerland

**Keywords:** cardiac resynchronization therapy, ECGI, His bundle pacing, His resynchronization therapy, noninvasive epicardial mapping

## Abstract

**Background:**

His bundle pacing (HBP) is an alternative to biventricular pacing (BVP) for delivering cardiac resynchronization therapy (CRT) in patients with heart failure and left bundle branch block (LBBB). It is not known whether ventricular activation times and patterns achieved by HBP are equivalent to intact conduction systems and not all patients with LBBB are resynchronized by HBP.

**Objective:**

To compare activation times and patterns of His‐CRT with BVP‐CRT, LBBB and intact conduction systems.

**Methods:**

In patients with LBBB, noninvasive epicardial mapping (ECG imaging) was performed during BVP and temporary HBP. Intrinsic activation was mapped in all subjects. Left ventricular activation times (LVAT) were measured and epicardial propagation mapping (EPM) was performed, to visualize epicardial wavefronts. Normal activation pattern and a normal LVAT range were determined from normal subjects.

**Results:**

Forty‐five patients were included, 24 with LBBB and LV impairment, and 21 with normal 12‐lead ECG and LV function. In 87.5% of patients with LBBB, His‐CRT successfully shortened LVAT by ≥10 ms. In 33.3%, His‐CRT resulted in complete ventricular resynchronization, with activation times and patterns indistinguishable from normal subjects. EPM identified propagation discontinuity artifacts in 83% of patients with LBBB. This was the best predictor of whether successful resynchronization was achieved by HBP (logarithmic odds ratio, 2.19; 95% confidence interval, 0.07–4.31; *p* = .04).

**Conclusion:**

Noninvasive electrocardiographic mapping appears to identify patients whose LBBB can be resynchronized by HBP. In contrast to BVP, His‐CRT may deliver the maximum potential ventricular resynchronization, returning activation times, and patterns to those seen in normal hearts.

**Abbreviations**
BVP: biventricular pacing
CRT: cardiac resynchronization therapy
ECG: electrocardiograph
ECGI: ECG imaging
HBP: His bundle pacing
His‐CRT: cardiac resynchronization therapy delivered by His bundle pacing
LBBB: left bundle branch block
LVAT‐95: left ventricular activation time of 95% of activations

## 1 INTRODUCTION

Left bundle branch block (LBBB) causes dyssynchronous ventricular activation.^1^ The aim of cardiac resynchronization therapy (CRT) is to correct this abnormality of electrical activation to improve cardiac function in patients with heart failure and LBBB. Biventricular pacing (BVP) is the most widely used method for delivering CRT and has been shown to improve symptoms, clinical outcomes, and mortality.^2^ However, many patients treated with BVP continue to experience high symptom burdens and poor prognoses.^3^ BVP is thought to produce its beneficial effect by delivering more synchronous ventricular activation and shortening atrioventricular interval.^4^

However, BVP does not completely correct the disordered ventricular activation that occurs with LBBB. In fact, it produces only relatively modest reductions in ventricular activation time and results in nonphysiological ventricular activation patterns.^1^ As a result, there has been interest in the development of more effective CRT. His bundle pacing (HBP) has recently been proposed as a method for delivering more effective ventricular resynchronization than BVP.^5^ His‐cardiac resynchronization therapy (His‐CRT) resynchronizes ventricular activation by overcoming proximal conduction system block, thereby activating the ventricles via the native His‐Purkinje system.^6^, ^7^ His‐CRT can produce greater QRS duration (QRSd) shortening and LVAT reduction than BVP, which translate to larger improvements in acute hemodynamic function.^3^

However, it does not appear to be possible to shorten ventricular activation time in all patients with 12‐lead ECG features of LBBB.^8^ HBP is most likely to deliver ventricular resynchronization in patients with proximal conduction system disease. The 12‐lead ECG is an imperfect tool for identifying the mechanism of conduction impairment. Upadyay et al. found, using intracardiac septal mapping, that conduction block within the left‐sided His fibers or proximal portion of the left bundle branch was present in 64% of patients with a 12‐lead ECG appearance of LBBB. These patients have the highest chance of successful resynchronization with HBP.^7^

A second challenge is that it is difficult, using the 12‐lead ECG, to quantify left ventricular resynchronization and therefore to determine whether the maximum potential resynchronization has been achieved: LVAT cannot be easily identified with the 12‐lead ECG. This can be particularly challenging in the presence of nonselective His bundle capture, which results in a pseudo‐delta wave as a result of local right ventricular myocardial capture at the lead tip.^3^ Furthermore, it is not known whether the reduction in activation times achieved with HBP is associated with restoration of the normal physiological ventricular activation pattern.^9^

Therefore, although His‐CRT is a promising alternative to BVP, before proceeding to long‐term head‐to‐head randomized control trials it would be helpful to develop tools for improving patient selection and intra‐procedural targets:

1) Noninvasive identification of patients in whom His‐CRT is likely to be successful would allow resynchronization strategy to be targeted to underlying conduction disorder.
2) Quantification of ventricular electrical resynchronization, with a normal range, will allow operators to easily establish whether ventricular resynchronization has been delivered and assess whether maximum activation time reduction has been realized.

We addressed these two questions by analysing the activation times and patterns of HBP, BVP, and intrinsic activation during LBBB and compared these to intact conduction systems using noninvasive epicardial activation and propagation mapping.

## 2 METHODS

### 2.1 Study population

Two groups of patients were recruited at a single tertiary cardiac center (Hammersmith Hospital, London, UK): (1) patients with LBBB and left ventricular impairment scheduled for biventricular pacemaker implantation with or without defibrillator function; (2) a comparison group of patients with normal 12‐lead QRS morphology and narrow QRSd undergoing epicardial mapping for ventricular ectopy ablation, arrhythmia risk stratification, or recruited specifically as normal subjects. Inclusion criteria for the CRT group were LBBB morphology on 12‐lead ECG with QRSd > 130 ms, EF < 35%, NYHA II–IV. Patients who were unable to give consent, or were clinically unstable, were excluded. Patients undergoing research protocols gave written, informed consent and the study was approved by the local ethics committee and health research authority (13/LO/1440, 10/LO/1660, and IRAS 258686). The 12‐lead surface ECG for each patient with LBBB was studied for adherence of the QRS morphology to pre‐defined LBBB criteria^10^ (AHA/ACC/HRS^11^ and Strauss et al.^12^). ECG criteria and further details of the recruited groups are provided in the Supporting Information.

### 2.2 Noninvasive epicardial electrical mapping (ECG imaging [ECGI])

All patients were fitted with a 252‐electrode ECGI vest (Medtronic) and underwent low dose thoracic computed tomography to acquire cardiac anatomy and electrode positions. The ECGI methodology has been described and validated previously^13^: multielectrode body‐surface potentials are combined with radiologically acquired anatomy using the ECGI solution to the inverse problem allowing reconstruction of unipolar epicardial electrograms (EGMs). For patients undergoing CRT, ECGI recordings were performed during AAI pacing (intrinsic ventricular activation), HBP, and BVP. For patients with normal QRS, ECGI recordings were made during sinus rhythm.

### 2.3 Activation time analysis

Activations from individual electrodes were temporally annotated based on the most negative dv/dt (the steepest slope of the voltage‐time relationship) and visualized on patients' three‐dimensional (3D) cardiac model. The total LVAT was calculated from the earliest to the latest activation. This value can be skewed by outliers caused by noise and anatomical/temporal mis‐annotation, therefore the activation time of 95% of activations (left ventricular activation time of 95% of activations [LVAT‐95]) was used to quantify resynchronization.

### 2.4 Epicardial propagation mapping (EPM)

To determine activation patterns, custom software was used to render movies of wavefront propagation across the epicardium. The entire duration of a single beat's EGM for each virtual epicardial electrode was visually represented on the 3D cardiac model. The EGM voltage was represented by circles moving outward from the cardiac model proportionate with the negative dv/dt with values <0 (i.e., positive dv/dt) clipped. By displaying the entire EGM, rather than the most negative dv/dt, visual interpretation was used to determine activation wavefronts rather than relying on potentially mis‐annotated activations.

### 2.5 Pacing

Temporary HBP was performed in patients with LBBB and was achieved via either the femoral or subclavian approach. If the femoral route was used, a quadripolar electrophysiology catheter was placed on the bundle of His. If the subclavian route was used, a SelectSecure 3830 lead was delivered via either a C304‐His deflectable sheath or C315 fixed curve sheath (leads and delivery system: Medtronic).^14^ The lead was not actively fixated unless BVP failed, in which case the SelectSecure 3830 lead was deployed for permanent HBP. Selective and nonselective His bundle capture was determined using standard criteria.^15^ BVP was performed using the standard technique and AAI pacing was performed from the right atrial appendage.

### 2.6 Statistical analysis

Patients were classified by the degree of resynchronization occurring with HBP. Successful resynchronization, referred to as His‐CRT, was defined as reduction in LVAT‐95 by 10 ms from intrinsic activation. This cut‐off was applied because we have previously demonstrated that the variation of LVAT‐95 in this setting is 10 ms^3^. Therefore, smaller reductions in LVAT‐95 may be due to measurement variation rather than true resynchronization. Failed His‐CRT was defined as a <10 ms reduction in LVAT‐95 compared to intrinsic activation (as long as 12‐lead ECG criteria for His bundle capture were fulfilled). His‐CRT was further subclassified into incomplete His‐CRT and complete His‐CRT. Complete His‐CRT was defined as HBP LVAT‐95 within the 99% range (mean ± 2.58 × *SD*) for intrinsic LVAT‐95 in patients with normal QRS. Incomplete His‐CRT was defined as patients with mean HBP shortening of LVAT‐95 by >10 ms but with mean HBP LVAT‐95 above the upper limit of normal defined by the 99% range for LVAT‐95 in patients with normal QRS. The unpaired *t* test was used to compare electrical parameters between groups, with paired *t* tests for within‐patient comparisons.

An ordinal regression model predicting the HBP LVAT (LVAT‐95) and including intrinsic LVAT‐95 was used to assess the impact of propagation pattern on the change in LVAT95. To present the data, the results of the ordinal regression model were transformed to produce LVAT‐95 times. Analyses were performed using the statistical environment “R,” using the package “rms.”^16^, ^17^

## 3 RESULTS

Forty‐six subjects were recruited, 25 with LBBB and left ventricular impairment, and 21 with narrow QRS and normal ventricular function. Due to technical error with the ECGI system, data was unavailable during HBP in one patient in the LBBB group. Therefore 24 patients with LBBB undergoing CRT device insertion were included alongside 21 subjects with normal ventricular function and narrow QRS. All 24 patients with LBBB underwent HBP and 20 had BVP ECGI recordings (due to unsuitable coronary sinus anatomy in four patients). Baseline demographics for both groups of patients are displayed in Table 1 and the flowchart for inclusion is found in Figure S1.

**Table 1:** Baseline characteristics

*Patients undergoing His bundle pacing (n = 24)*	
Age, years	68 ± 10 (48 – 89)
Male	15 (63%)
Ejection fraction (%)	27 ± 8 (14 – 42.5)
NYHA functional class	2.3 ± 0.7 (1 – 4)
I	1 (4%)
II	16 (67%)
III	5 (21%)
IV	2 (8%)
Previous MI	10 (42%)
ACE inhibitor/ARB	23 (96%)
β‐Blocker	21 (88%)
MRA	16 (67%)
Sacubitril	2 (8%)
QRS duration (ms)	
Intrinsic activation	173 ± 15 (136 – 208)
His bundle pacing	144 ± 27 (106 – 184)
Biventricular pacing^a^	157 ± 21 (109 – 195)
PR interval (ms)	195 ± 50 (130 – 384)
Selective His bundle capture	2 (8%)

*Patients with narrow QRS (n = 21)*	
Age, years	43 ± 12 (25 – 68)
Male	14 (67%)
Ejection fraction (%)	57 ± 5 (49 – 75)
NYHA functional class	1.1 ± 0.3 (1 – 2)
I	19 (90%)
II	2 (10%)
Previous MI	0 (0%)
QRS duration (ms)	98 ± 8

Abbreviations: CS, coronary sinus; MRA, mineralocorticoid receptor antagonist.

*Note*: Values are mean ± *SD* (range) or *n* (%). In one patient of patients with left bundle branch block undergoing His bundle pacing, the ejection fraction measured at referral was higher than 35%. One patient in the group of patients with a narrow intrinsic QRS had an ejection fraction of 49%, which is in the mildly impaired category but the assessment clinically was low‐normal function.

^a^ *n* = 21.

### 3.1 Activation times

**Figure 1:**
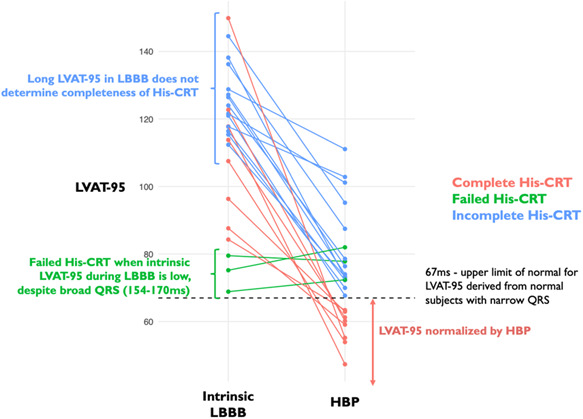
LVAT‐95 times during intrinsic rhythm and HBP in patients with LBBB. The dotted line represents the upper limit of normal (70 ms) derived from subjects with normal, narrow QRS complexes. Patients in green did not shorten LVAT‐95 by at least 10 ms with HBP (failed His‐CRT). Patients in blue shortened LVAT‐95 by at least 10 ms with HBP but did not achieve LVAT‐95 in the normal range. Patients in red shortened LVAT‐95 by at least 10 ms with HBP and achieved a LVAT‐95 within the normal range. CRT, cardiac resynchronization therapy; HBP, His bundle pacing; LBBB, left bundle branch block; LVAT‐95, left ventricular activation time of 95% of activations

The activation times in intrinsic rhythm, HBP, and BVP are displayed in Table 2. Figure 1 displays the distribution and degree of LVAT‐95 shortening of patients who achieved complete, incomplete, and failed His‐CRT. We defined complete His‐CRT as LVAT‐95 within the normal range defined by subjects with normal QRS. Signal‐to‐noise ratio (measured as the mean difference from intrinsic LBBB to His‐CRT divided by the *SD* of this mean difference) was higher for LVAT‐95 (−1.69) as compared to QRSd (−1.22).

**Table 2:** Activation times

Parameter				LVAT‐95 (ms)			*p* value		
Intrinsic, unpaced LBBB				113 (104–123)			
Intrinsic narrow QRS				47 (28–67)^a^					
Difference between the above groups				67 (57–76)			<.0001		
HBP (in patients with LBBB)				74 (67–81)					

His‐CRT type	HBP LVAT‐95 shortening (from intrinsic LBBB)			HBP LVAT‐95 shortening (from BVP)			BVP LVAT‐95 shortening (from intrinsic LBBB)		
	*N* (%)	∆LVAT‐95 (ms)	*p* value	*N*	∆LVAT‐95 (ms)	*p* value	∆LVAT‐95 (ms)		*p* value
All patients	24	40 (29 – 50)	<.0001	20	27 (16 – 38)	.000242	9 (−2 – 20)		.167
Complete His‐CRT	8 (33.3)	52 (32 – 72)	.0005	7	40 (27 – 53)	.013	6 (−8 – 19)		.671
Incomplete His‐CRT	13 (54.1)	42 (33 – 51)	<.0001	10	17 (6 – 28)	.0589	25 (17 – 32)		<.0001
Failed His‐CRT	3 (12.5)	n/a	n/a^b^	3	30 (19 – 41)	.0516	−33 (−22 – ‐44)		.0431

*Note*: Values are mean ± 95% confidence interval except for intrinsic LVAT‐95 in narrow complex patients where normal range (99% range) is provided.

Abbreviations: BVP, biventricular pacing; CRT, cardiac resynchronization therapy; HBP, His bundle pacing; LBBB, left bundle branch block; LVAT‐95, left ventricular activation time of 95% of activations.

^a^ Normal range (99% range).

^b^ By definition <10 ms shortening.

### 3.2 Activation patterns

Visual analysis of ventricular propagation maps identified revealed two distinct patterns of LV activation during intrinsic LBBB. In the majority of patients (20/24, 83.3%), there were appearances of regions of epicardial propagation block, which we term “propagation discontinuities.” In most of these patients (17/20, 85%) propagation discontinuities manifested as distinct “lines of block,” where wavefront propagation appeared to halt simultaneously along a line. Beyond the line of discontinuity, epicardium appeared to be activated from a different direction than the original propagation wavefront. This was not necessarily the opposite direction: the wavefronts could be orthogonal resulting in the appearance of “U‐shaped” activation. In the remaining 15%, epicardial propagation discontinuity was instead observed as “zones of slow conduction,” where epicardium beyond the line of block appeared to be activated, after a delay, in the same direction that the original wavefront was traveling in. Two patients displayed more than one line of discontinuity (anterior and posterior). The majority of lines of discontinuity were longitudinal (17/22, 77%) and found on the anterior LV (14/22, 64%) with fewer found on the anterolateral LV (6/22, 27%) and the remaining discontinuities visualized on the posterior LV (2/22, 9%). They were seen in patients with ischemic and nonischemia etiology of heart failure.

**Figure 2:**
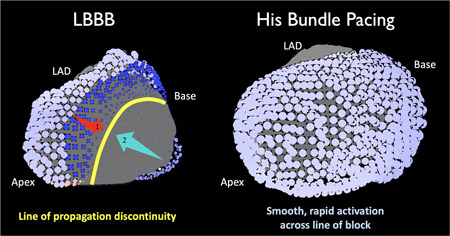
Example of propagation discontinuity and successful correction by HBP. The epicardial surface of the LV is shown for intrinsic LBBB (left) and HBP with shortening of LVAT (right) in the same patient. Blue circles are ECGI derived activations. Darker blue represents later activation. In LBBB, the activation wavefront appears to initially travel from the anterior LV toward the lateral LV (arrow 1, red) but then appears to halt at a line of propagation discontinuity (yellow). The remainder of the LV appears to be activated later from the posterior LV (arrow 2, blue). During HBP, the wavefront moves smoothly and rapidly across the line of propagation discontinuity. ECGI, ECG imaging; HBP, His bundle pacing; LBBB, left bundle branch block; LV, left ventricle; LVAT, left ventricular activation time

In the remainder of patients with a 12‐lead appearance of LBBB (4/24, 16.7%), lines of block were not observed. Instead, a second pattern was observed: slow propagation across the LV epicardium without any appearance of discontinuity. This was termed diffusely slowed propagation (DSP). Examples of both patterns are shown in *accompanying videos* and illustrative single‐frames of EPM cines are shown in Figure 2.

During HBP, in 15 (75%) of the 20 patients where propagation discontinuity was observed, the appearance of discontinuity resolved with rapid, unidirectional activation proceeding through the regions where propagation discontinuity was observed in intrinsic activation. In the remaining five patients, the appearance of discontinuity was preserved. In patients without the appearance of discontinuity, HBP produced an activation pattern of slow, but unblocked, propagation across the LV epicardium consistent with intrinsic activation. EPM maps of BVP in patients with LBBB showed collision of slowly propagating wavefronts on the LV epicardium. All patients with narrow QRS showed rapid, smooth activation of the LV epicardium without any appearance of block or regionally slowed conduction. All patients who displayed complete His‐CRT (HBP LVAT‐95 within the normal range defined by subjects with narrow QRS), displayed resolution of the propagation discontinuity and the LV activation pattern was indistinguishable from subjects with a normal QRS.

Where regional discontinuity was seen to disappear with HBP yet incomplete His‐CRT occurred, the change in propagation pattern from intrinsic LBBB to HBP showed that lines of propagation discontinuity in some regions were intact with HBP while others disappeared, allowing postulation of mechanisms of incompleteness of His‐CRT. For example, in two patients the basal portion of a longitudinal line of discontinuity disappeared with HBP with the apical portion left intact. This suggests selective recruitment of the anterior fascicle of the left bundle but failure to recruit the posterior fascicle (demonstrated in the *accompanying videos*).

### 3.3 Predictors of successful His‐CRT

#### 3.3.1 Appearance of propagation discontinuity

**Figure 3:**
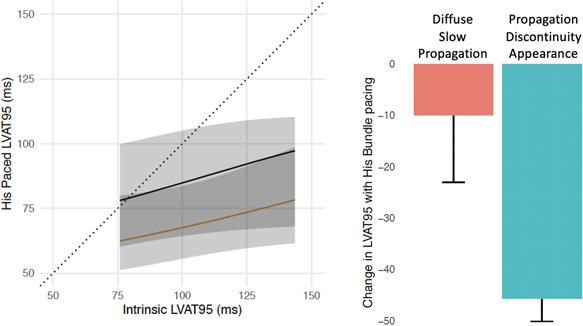
Impact of propagation discontinuity during intrinsic conduction in LBBB on change of LVAT with HBP. (Left) Ordinal regression of HBP LVAT‐95 from intrinsic LVAT‐95 in patients with LBBB. The black line represents patients with diffuse slow propagation (absence of propagation discontinuity) in LBBB. The yellow line represents patients with regions of propagation discontinuity in LBBB. Patients with propagation discontinuity were more likely to achieve a shorter LVAT‐95. (Right) change in LVAT‐95 from intrinsic LBBB to HBP in patients without (red) and with (blue) appearance of propagation discontinuity in LBBB. HBP, His bundle pacing; LBBB, left bundle branch block; LVAT‐95, left ventricular activation time of 95% of activations

When propagation discontinuity was seen in intrinsic LBBB, HBP was much more likely to result in a shorter LVAT‐95 than if propagation discontinuity was absent (logarithmic odds ratio (logOR), 2.19; 95% confidence interval [CI], 0.07–4.31; *p* = .04). For ease of comprehension, we also used the presence of discontinuity as a dichotomized positive test and successful His‐CRT (LVAT‐95 shortening by at least 10 ms) as a dichotomized positive outcome of interest. In this model, the power of LVAT‐95 shortening as a continuous value is lost in favor of ease of comprehension: for the prediction of successful His‐CRT, the presence of propagation discontinuity has a specificity and positive predictive value of 100% and a sensitivity of 95% with a negative predictive value of 75%. When propagation discontinuity was resolved by HBP, HBP was much more likely to result in a shorter LVAT‐95 than if propagation discontinuity remained present during HBP or discontinuity was not present (logOR, 1.89; 95% CI, 0.21–3.56; *p* = .03; Figure 3).

#### 3.3.2 LVAT during intrinsic activation

In every patient in which HBP failed to shorten LVAT (failed His‐CRT), intrinsic LVAT‐95 was <80 ms. These short intrinsic LVAT‐95 times occurred despite the presence of a broad 12‐lead ECG QRS (154–170 ms; Figure 1). In all patients where the intrinsic LVAT‐95 was >80 ms, HBP resulting in at least partial resynchronization (>10 ms LVAT‐95 shortening). As continuous variables, however, intrinsic LVAT‐95 did not predict the degree of LVAT‐95 shortening.

#### 3.3.3 Twelve‐Lead ECG

When the full set of Strauss criteria for LBBB were met, HBP was much more likely to result in a shorter LVAT‐95 than if Strauss criteria were not fully met (logOR, 1.59; 95% CI, −0.13–3.31; *p* = .07). The predictive ability was not as high as that of propagation discontinuity. If the full set of AHA/ACC/HRS criteria for LBBB were met, HBP was less likely to result in a shorter LVAT‐95 than if the criteria were not met (logOR −0.46; 95% CI, −1.99–1.07; *p* = .06).

## 4 DISCUSSION

In this study we demonstrate the novelty and utility of analysing activation patterns and times of His‐CRT for LBBB in several ways:

(1) EPM is a novel tool for noninvasively assessing ventricular activation patterns.
(2) The activation pattern in LBBB can identify patients in whom HBP is likely to result in ventricular resynchronization.
(3) Noninvasive electrical mapping can quantify ventricular resynchronization and identify mechanisms of incomplete resynchronization.
(4) Successful His‐CRT results in normalization of ventricular activation with a more physiological activation pattern than BVP.

### 4.1 LBBB activation patterns

We used noninvasive mapping to identify two distinct types of the ventricular activation patterns in patients with 12‐lead ECG appearances of LBBB: those with the appearance of regions of propagation block, which we term “propagation discontinuity” and those with slow propagation without regions of discontinuity. Our findings are consistent with those from previous studies investigating ventricular activation patterns during LBBB, using both endocardial and epicardial mapping.^18^, ^19^, ^20^, ^21^ Auricchio et al.^18^ observed through endocardial noncontact mapping, as we did through EPM, that the vast majority of block is observed on the anterolateral portion of the LV (90% in this study compared to 87% in Auricchio et al.). The correction of these propagation discontinuities by HBP supports the notion that this block is functional in character and furthermore suggests that propagation discontinuities are the epicardial manifestation of block within the proximal conduction system.

The presence of this proximal conduction system block appears to be a useful predictor of whether HBP is likely to be successful in delivering ventricular resynchronization. Upadhyay et al.^7^ demonstrated proximal conduction system block in either the His bundle or the left bundle branch in 64% of patients with LBBB undergoing mapping of the left‐sided conduction system, 85% of whom demonstrated HBP correction of LBBB. In the remaining 36% of patients, the proximal conduction system appeared intact despite ECG appearance of LBBB, none of whose LBBB was corrected by HBP. Therefore the presence of epicardial propagation discontinuity appears to identify people who have proximal conduction system disease, which can be corrected by HBP.

### 4.2 Conduction system block versus propagation discontinuity

It is important to conceptually and anatomically distinguish disease within the His‐Purkinje conduction system from the appearance of propagation discontinuity on the LV epicardial surface. Upadhyay et al.'s^7^ observations are in keeping with the most plausible model for LBBB correction by HBP, where functional proximal conduction block is bypassed by delivering energy distal to the site of block to recruit latent, viable conduction fibers. The appearances of propagation discontinuity we observed in our study were not isolated to proximal sites near the His bundle, despite resolving with HBP, and the orientation was variable: both longitudinal and transverse. We propose that this is because EPM visualizes myocardial activation rather than conduction system activation. Myocardial activation is determined by upstream conduction‐system behavior. Thus the appearance of epicardial propagation discontinuity is a downstream myocardial manifestation of conduction‐system block. This explanation equates propagation discontinuity with conduction system block and equates DSP with intact Purkinje activation. This is supported by our finding that no patients with a diffusely slowed conduction pattern corrected LBBB with HBP while patients with propagation discontinuity frequently corrected, with the noninvasive mapping pattern showing similar predictive ability to septal mapping.^7^ It remains unclear whether patients with block too distal to be corrected by HBP would display appearances of lines of propagation discontinuity or DSP, since such patients are indistinguishable from patients with intact Purkinje activation in this study.

Propagation discontinuity is not observed on direct endocardial contact mapping,^18^ indicating the specific pattern of slowed or blocked propagation may be an artifact produced by the ECGI system's inverse solution^22^ (which is also supported by in silico modeling^23^) or an artifact arising from epi‐endo anisotropy. Importantly our study shows that these block artifacts correspond to biological phenomena due to their ability to identify patients amenable to resynchronization. We term the appearance discontinuity, rather than block, to differentiate the appearance we observed, that might be artifactual, from the specific electrophysiological definition of “block.”

### 4.3 Clinical utility of ECGI derived measures for His‐CRT

Regardless of their pathophysiological basis, ECGI‐derived activation patterns in LBBB allow accurate, noninvasive prediction of patients whose LVAT‐95 is shortened by HBP. We have previously demonstrated that LVAT‐95 shortening is a key mechanism through which successful His‐CRT improves cardiac output, with incremental activation time shortening correlating with hemodynamic improvement.^3^ Although left‐sided septal conduction system mapping also appears to be a powerful predictor of HBP response,^7^ the invasive nature of the technique precludes routine clinical use. The baseline 12‐lead QRSd is less reliable at predicting HBP resynchronization and EPM provides superior predictive ability to 12‐lead QRS morphology analysis: the Strauss criteria's predictive ability trended toward significance in this study but the degree of resynchronization predicted with this was much lower than with ECGI appearance of propagation block. Our study provides evidence that it is technically feasible to improve patient selection for His‐CRT using noninvasive propagation mapping. We have also shown that the predictive feature of block artifact can be observed even when a different patient's anatomy is used to analyse propagation. Therefore it is likely that simpler noninvasive mapping methods which require fewer electrodes and eliminating the need for CT imaging could be develop, facilitating widespread use in clinical practice.^24^, ^25^

LVAT‐95 also provides an intra‐procedural target with complete His‐CRT representing activation normalization that is not affected by selectivity of His bundle capture and with superior signal‐to‐noise ratio than QRSd. QRSd is affected by capture selectivity (Figure S2) but subtraction of Stim‐endQRS time from His‐endQRS time is an alternative 12‐lead ECG surrogate for electrical resynchronization that, like LVAT‐95, also ignores selectivity of His bundle capture. However, LVAT‐95 shortening correlates with hemodynamic response and avoids arbitrary and variable QRSd offset measurement and mistaken inclusion of HV shortening into resynchronization. Where the ECGI technology is not available, our findings show that the next best alternative is to ensure that only patients meeting full Strauss LBBB 12‐lead ECG criteria are selected for His‐CRT. The presence of lateral notching and anterior QS/rS appear to be important markers of conduction system disease amenable to HBP, whereas the lateral monophasic R wave requirement of the ACC/AHA/HRS guidance does not appear to be predictive. Similar discrepancies between the two criteria have been reported for BVP response.^12^ This is not surprising as the activation pattern of “true” LBBB with posterolateral late activation, due to anterior propagation block, will also be more amenable to coronary sinus leads.

The HIS‐SYNC randomized pilot evaluation of His‐CRT vs BVP for heart failure with LBBB did not demonstrate the superiority of His‐CRT over BVP.^8^ The authors suggest that a 50% crossover from the His‐CRT arm to the BVP arm was due to two factors: a current lack of reliably successful HBP tools and, crucially, patient selection. It was felt that many patients enrolled who did not successfully resynchronize with HBP actually had a form of nonspecific intraventricular conduction delay (IVCD) rather than LBBB and even in those with ECG‐diagnosed LBBB, conduction system block may not have been the mechanism of LBBB as this could not be elucidated from the 12‐lead ECG. These patients crossed over the BVP arm as they could not be resynchronized. Their on‐treatment results, however, were very promising and corroborate our own previous findings that when resynchronization occurs there is scope for clinically important improvements in hard outcomes. Using noninvasively measured EPM propagation discontinuity for patient selection and LVAT‐95 as a target measure for resynchronization could transform the feasibility of His‐CRT and permit a randomized controlled trial with a high success rate in the His‐CRT arm. The appropriate taxonomy for patients with 12‐lead ECG appearances of LBBB that do not fulfill the full Strauss criteria, do not correspond to epicardial propagation dicontinuity or have intact Purkinje activation on septal mapping may be IVCD but to maintain consistency with historical LBBB diagnoses they may be referred to as atypical LBBB or LBBB without features conducive to resynchronization.

### 4.4 Physiological resynchronization

HBP has been hailed as a method for physiological resynchronization due to the normalized appearance and short duration of the QRS complex that are often seen as well as the purported mechanism of recruitment of the left bundle which would then activate the ventricles physiologically. However, QRSd and morphology are not reliable measures of resynchronization by HBP, particularly as selectivity of His bundle capture affects both. We have, for the first time, defined a normal range for LVAT and have found that when HBP produces activation times within this range, left ventricular activation pattern is indistinguishable from normal left ventricular activation. Both produce rapid, smooth, unimpeded activation of the left ventricle. This suggests that HBP has the potential to fulfill the maximum potential of CRT. Alongside Upadhyay et al.'s^7^ observation of restoration of Purkinje potentials there is now compelling evidence that HBP does indeed produce physiological resynchronization akin to normal LV activation.

In contrast, EPM reveals the nonphysiological character of BVP. Wavefronts emanate from the positions of the right and left ventricular electrodes traveling at slow myocardial conduction speeds rather than rapid conduction‐system speed. These wavefronts then collide on the surface of the left ventricle. Although regions of conduction block are not observed, they are replaced by lines of wavefront collision. We have previously shown that the within‐patient incremental activation shortening seen with His‐CRT over BVP closely correlates with the incremental hemodynamic improvement. However, the correlation is not perfect: further hemodynamic benefits are seen with HBP over BVP than anticipated by LVAT‐95 shortening alone.^3^ The physiological nature of His‐CRT, and the contrasting nonphysiological nature of BVP, may account for the extra benefit of HBP, providing further justification of the potential role for His‐CRT as a superior alternative to BVP.

### 4.5 Partial and failed correction of LBBB

In three patients HBP did not improve LVAT‐95 by at least 10 ms “failed His‐CRT” and 13 patients reduced LVAT by more than 10 ms but did not achieve LVAT‐95 within the normal range defined in this study. We have previously demonstrated that even when no resynchronization occurs there is still scope for hemodynamic improvement through AV optimization.^26^ In patients with partial HBP correction of LBBB (incomplete His‐CRT), the within‐patient incremental LVAT‐95 improvement with HBP over BVP was 17 ms, suggesting that even incomplete His‐CRT, without activation normalization, produces superior electrical resynchronization to BVP. Propagation mapping also allows us to postulate mechanisms incompleteness of His‐CRT in this group. Where lines of discontinuity in regions, whose conduction system supply is the anterior fascicle, resolve with HBP but posterior portions are left intact we can infer that selective recruitment of the anterior fascicle has occurred. In such cases, propagation mapping can guide the operator to attempt pacing more distally to attempt posterior fascicle capture either in the distal His bundle or the left bundle area.

### 4.6 Epicardial propagation mapping

This study introduces EPM as a novel method for noninvasively assessing activation patterns. Conventional activation maps that annotate an activation time at most negative dv/dt are prone to potential mis‐annotation of activation when electrodes' EGMs show multiple waves. This occurs in bundle branch block but EPM visualizes the entire EGM so that human analysis can determine the true wavefront. This provides a powerful tool for accurately depicting patterns of activation that makes use of all electrical information acquired by ECGI systems.

### 4.7 Limitations

ECGI‐derived measures provide information on epicardial activation; the septum, where the conduction system lesion is likely to be located, is thus not analysed. However, LVAT‐95 measures the downstream effect of conduction system behavior in the septum on the LV myocardium overall. Furthermore, our findings are consistent with those produced by septal mapping of the conduction system and LVAT‐95 correlates with hemodynamic outcomes.^3^ Nevertheless, there are other limitations of the ECGI methodology including the assumption of static geometry and validity of particular solution to the inverse problem employed by ECGI. The hemodynamic changes predicted by LVAT‐95 are acute and longitudinal evidence is required to assess the long‐term clinical correlates of LVAT‐95. As discussed above, it has been suggested that ECGI appearances of lines of discontinuity may in fact be artifactual due to the methodology of ECGI.^27^ However, the elimination of lines of discontinuity correlate with shorter activation time suggesting biological electrophysiological phenomena. Although the appearance of lines of discontinuity are stark, they currently require human visual interpretation and thus may be subject to a degree of inter‐ and intra‐rater irreproducibility. Operator experience can affect the success of His‐CRT to confound prediction of successful His‐CRT, however, by using very high output temporary HBP (up to 25 mA) we maximized the chance of correcting LBBB even if the position was not optimal.

## 5 CONCLUSION

EPM derived from ECGI allows accurate noninvasive discrimination of LBBB with regions of propagation discontinuity that is potentially amenable to resynchronization by HBP from diffuse slow conduction that cannot be corrected. When HBP normalizes LVAT, the activation pattern produced is physiological and indistinguishable from normal activation with intact conduction system.

## Supporting information

Supporting information.

Supporting information.

## Data Availability

The data set for this study is not available to third parties as ethical approval did not include this.
