# Supplementary material for: Electrocardiographic predictors of successful resynchronization of left bundle branch block by His bundle pacing
Source: J Cardiovasc Electrophysiol. 2021 Jan 4;32(2):428–38. doi: 10.1111/jce.14845 (PMC8607473; doi:10.1111/jce.14845)
Supplement: Supplementary file 1 — Supporting information. [file JCE-32-428-s001.docx]

Online Appendix

# **Supplementary Figures**

| **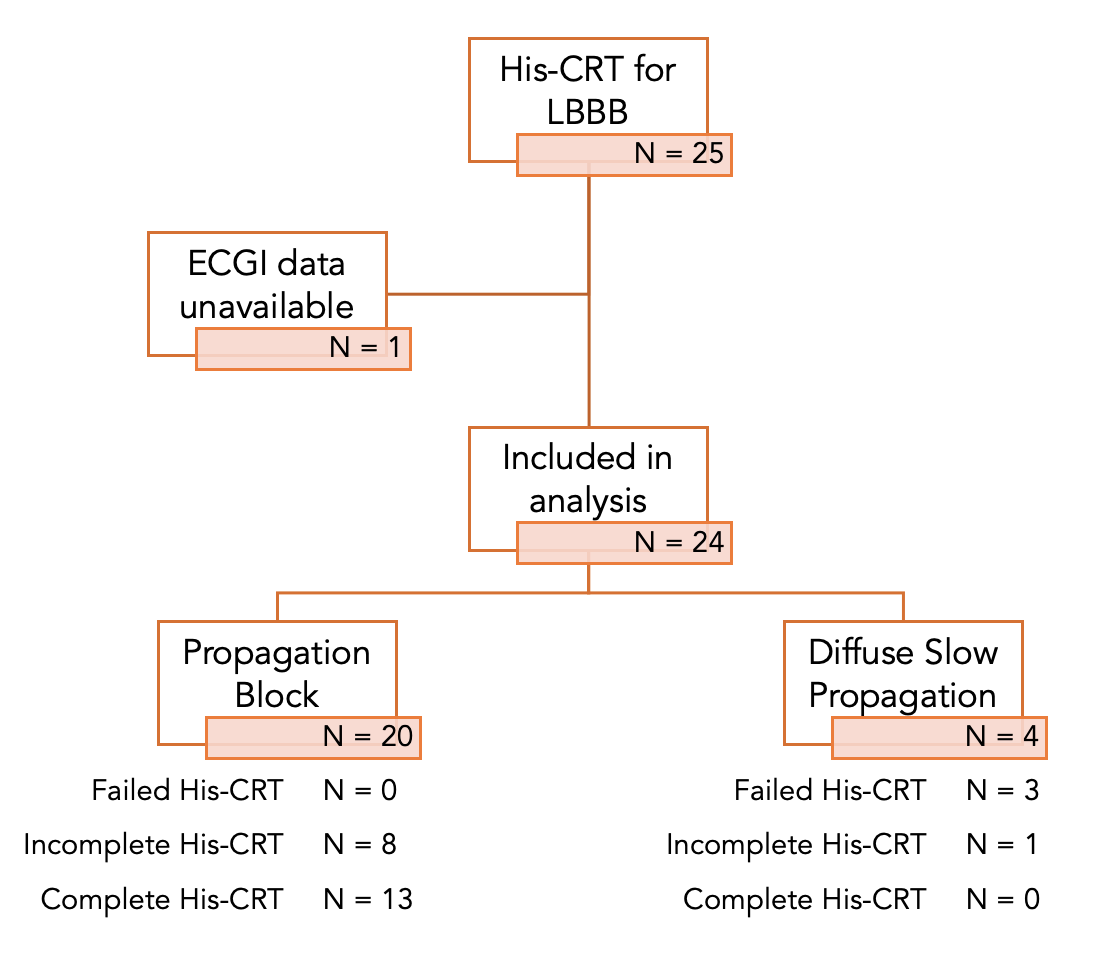** |
| --- |
| **Supplementary Figure S1**. **Flow chart of patients included in study.**  Of the 20 patients with propagation block, 3 displayed zones of slow conduction rather than the appearance of absolute block. In 2 such patients incomplete His-CRT was observed and in one complete His-CRT was observed. ECGI – ECG Imaging; LBBB – Left Bundle Branch Block; His-CRT – His Resynchronization Therapy; EPM -Epicardial Propagation Mapping |

| **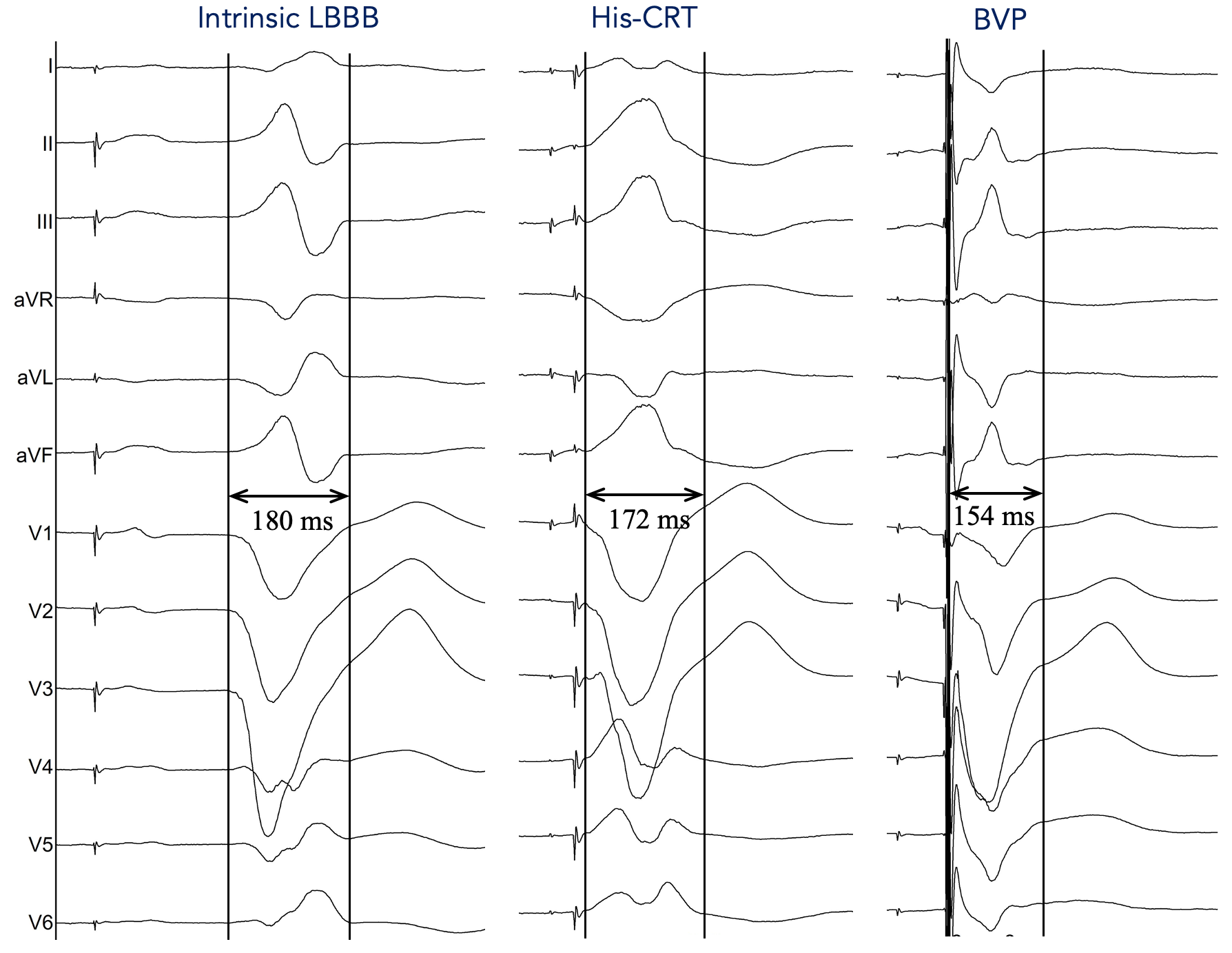** |
| --- |
| **Supplementary Figure S2: Incomplete His-CRT**  12-lead ECGs of intrinsic LBBB, non-selective HBP and BVP in a patient with EPM appearance of a line of propagation block in LBBB that disappears with HBP. Intrinsic QRS morphology meets Strauss criteria for LBBB with long QRSd (182ms), anterior QS and lateral notching but AHA/ACC/HRS criteria are not met due to lateral q waves. 12-lead QRS analysis suggests that BVP (26 ms QRSd shortening) has produced greater electrical resynchronization than HBP (8 ms QRSd shortening). ECGI analysis reveals that HBP has produced 49 ms LVAT-95 shortening from intrinsic LBBB. HBP has resulting in a LVAT-95 of 95.2 ms, which is above the upper limit of normal but the amount of LVAT-95 shortening is nevertheless substantial: incomplete His-CRT has occurred. BVP produces just 37.2 ms LVAT-95 shortening. QRS duration and morphology is less useful than LVAT-95 for measuring left ventricular electrical resynchronization due to the effect of non-selective His capture on early QRS and the higher resolution of ECGI.  LBBB – Left bundle branch block; HBP – His bundle pacing; BVP - biventricular pacing; His-CRT – Cardiac Resynchronisation Therapy by His bundle pacing; LVAT-95 – left ventricular activation time (95%); QRSd – QRS morphology. |

# **Supplementary Methods**

**ECG definitions**

The 12-lead surface ECG for each patient with LBBB was studied for adherence of the QRS morphology to pre-defined LBBB criteria(1). Both the AHA/ACC/HRS(2) criteria (which are 1) QRSd ≥120 ms, 2) QRS notching or slurring at least two of leads I, aVL and V5-6 and 3) monophasic R wave, without q wave, in leads I and V5-6 and 4) R wave peak time > 60 ms in V5-6) and the so-called ‘Strauss(3) criteria’ (which are 1) QRSd ≥130 ms in women, ≥140 in men, 2) QRS notching or slurring in at least two of leads I, aVL, V1-2 and V5-6 and 3) QS or rS in leads V1-2) for LBBB were analysed.

**Recruited Subjects**

Patients in the left bundle branch block (LBBB) group referred for cardiac resynchronization therapy (CRT) were included if their ECG showed a QRSd of at least 130ms and was designated LBBB morphology by the referring physician (as well as meeting non-ECG criteria for CRT) and specifically consented to participate in the research protocol (13/LO/1440). These patients include the 18 successful His-CRT patients recruited in our previous analysis(4) and 3 further successful His-CRT patients not included in that study due to failed BVP. BVP was performed at a range of AV delays with a VV offset of zero with quadripolar lead vectors in the positions used for chronic BVP.

Although not all patients met AHA/HRS/ACC or Strauss criteria, such patients are referred to as patients with 12-lead ECG features of LBBB. The taxonomy of ECGs that do not meet full AHA/HRS/ACC or Strauss criteria is unclear. These patients can be referred to as displaying non-specific intraventricular conduction delay (IVCD) but may also be considered to be variants of the 12-lead ECG LBBB appearance. Indeed even when full Straus and AHA/HRS/ACC criteria are met, LBBB is not always reversed by His bundle pacing even at high output implying that designating all such patients “true” LBBB may not be accurate.

Subjects with normal, narrow QRS complex on 12-lead ECG and normal LV function comprised of two groups. Firstly, patients who underwent ECGI guided ablation of ventricular ectopy were included. These patients were not consented for a research protocol but retrospective ethical approval was sought to access their anonymised data for research purposes (IRAS 258686). Secondly, subjects who are relatives of patients with confirmed diagnoses of inherited arrhythmia syndromes (eg Brugada syndrome). These subjects were undergoing routine clinical screening for such syndromes but were also specifically consented to undergo ECGI recordings as part of a research protocol (10/LO/1660). Subjects were included if they were subsequently not found to have an inherited arrhythmia syndrome. However, it is possible that despite an arrhythmic diagnosis being 'excluded' they might still be carriers of as-yet-unrecognised mutations that affect their activation pattern or activation time (undetected by the 12-lead ECG).

# **Online Videos 1-6**

6 online videos are submitted. The caption for videos 1-6 are as follows. The videos are compiled into a single file and played in this order.

1. **Intrinsic Left Bundle Branch Block – Appearance of Propagation Block**

These are examples of epicardial propagation mapping. A single beat’s activation is displayed in each video. Coloured circles on the ventricular surface grow in size over time in proportion with the amplitude of the voltage of the electrogram (EGM) at that position. Blue circles are later activations and red circles are earlier activations. Therefore the progression of rapidly enlarging circles depicts a wavefront of activation propagating across the ventricular surface. Video 1 shows an example of unpaced activation in a patient with intrinsic left bundle branch block (LBBB). The left ventricular (LV) surface is visualised with the left anterior descending (LAD) coronary artery demarcating the anterior border of the LV. A wave of propagation proceeds from the LAD towards the mid-LV but halts longitudinally (marked in yellow). The remainder of the LV appears to be activated from the posterior LV. This appearance is referred as a propagation discontinuity.

1. **His bundle pacing - Correction of Left Bundle Branch Block**

This is a video of the same patient seen in video 1 but this video is an example of His bundle pacing, which has corrected LBBB. LV activation is rapid and smooth, without any evidence of the propagation discontinuity seen in intrinsic LBBB.

1. **Intrinsic Narrow QRS – Rapid Left Ventricular Activation**

This is a subject with narrow QRS and normal LV function. LV activation is rapid and smooth, similar to His bundle pacing seen in video 2.

1. **Intrinsic Left Bundle Branch block – Diffuse Slow Propagation**

This is a different patient, with an example of a beat of intrinsic LBBB shown. There is no appearance of block in this patient’s propagation map. Instead the less common appearance of diffusely slowed propagation is seen.

1. **Intrinsic Left Bundle Branch Block – Appearance of Propagation Block**

This is a different patient during intrinsic LBBB. Propagation block is observed.

1. **His Bundle Pacing – Appearance of Uni-fascicular Recruitment**

This is a video of the same patient seen in video 5 but this video is an example of His bundle pacing, which has only partially corrected LBBB. The mid-apical propagation block is still evident but the basal propagation block is no longer evident with early activation in the basal anterior and anterolateral LV activated early and from the septum rather than late and from the posterior LV, which occurred in intrinsic LBBB. We can infer that only some of the left bundle fibres have been recruited, potentially the anterior fascicle, while some remain blocked.

# References

1. Caputo ML, van Stipdonk A, Illner A et al. The definition of left bundle branch block influences the response to cardiac resynchronization therapy. International journal of cardiology 2018;269:165-169.

2. Surawicz B, Childers R, Deal BJ, Gettes LS. AHA/ACCF/HRS recommendations for the standardization and interpretation of the electrocardiogram: part III: intraventricular conduction disturbances a scientific statement from the American Heart Association Electrocardiography and Arrhythmias Committee, Council on Clinical Cardiology; the American College of Cardiology Foundation; and the Heart Rhythm Society endorsed by the International Society for Computerized Electrocardiology. Journal of the American College of Cardiology 2009;53:976-981.

3. Strauss DG, Selvester RH, Wagner GS. Defining left bundle branch block in the era of cardiac resynchronization therapy. The American journal of cardiology 2011;107:927-934.

4. Arnold AD, Shun-Shin MJ, Keene D et al. His resynchronization versus biventricular pacing in patients with heart failure and left bundle branch block. Journal of the American College of Cardiology 2018;72:3112-3122.
